# Genomics and justice: mitigating the potential harms and inequities that arise from the implementation of genomics in medicine

**DOI:** 10.1007/s00439-022-02453-w

**Published:** 2022-04-12

**Authors:** A. J. Clarke, C. G. van El

**Affiliations:** 1 Division of Cancer & Genetics, School of Medicine, Cardiff University, Wales, UK; 2 Department of Human Genetics and Amsterdam Public Health Research Institute, Amsterdam UMC, Vrije Universiteit Amsterdam, Amsterdam, The Netherlands

## Abstract

Advances in human genetics raise many social and ethical issues. The application of genomic technologies to healthcare has raised many questions at the level of the individual and the family, about conflicts of interest among professionals, and about the limitations of genomic testing. In this paper, we attend to broader questions of social justice, such as how the implementation of genomics within healthcare could exacerbate pre-existing inequities or the discrimination against social groups. By anticipating these potential problems, we hope to minimise their impact. We group the issues to address into six categories: (i) access to healthcare in general, not specific to genetics. This ranges from healthcare insurance to personal behaviours. (ii) data management and societal discrimination against groups on the basis of genetics. (iii) epigenetics research recognises how early life exposure to stress, including malnutrition and social deprivation, can lead to ill health in adult life and further social disadvantage. (iv) psychiatric genomics and the genetics of IQ may address important questions of therapeutics but could also be used to disadvantage specific social or ethnic groups. (v) complex diseases are influenced by many factors, including genetic polymorphisms of individually small effect. A focus on these polygenic influences distracts from environmental factors that are more open to effective interventions. (vi) population genomic screening aims to support couples making decisions about reproduction. However, this remains a highly contentious area. We need to maintain a careful balance of the competing social and ethical tensions as the technology continues to develop.

## Introduction

The young science of genomics started as a technical development—the rapid sequencing of an organism’s DNA—but has become a new science. Its greater speed of analysis does not merely provide answers more rapidly but enables new questions to be asked. Through an understanding of genetic difference achieved by genomics, it becomes possible to examine afresh the influence of environmental and life-course factors across the whole of biology. In relation to human healthcare, it opens up the understanding of disease. This, one hopes, will lead to interventions to prevent a broad range of conditions and to treat what cannot be prevented.

Raising such hopes, however, also raises a set of questions, including the extent to which the promises are justified and who will benefit from them. This actually comprises two questions, i.e. (i) who will benefit from the advances in understanding and treating disease and (ii) who will benefit from the promises, whether or not they work out. As well as the lives of patients, the careers of scientists and the expansion of commercial enterprises are at stake. When these interests do not align, who or what will win out?

When ethical questions arise in relation to the clinical applications of genetics and genomics, the concerns are more often framed in terms of consent and understanding, or the making of decisions by a patient about testing, informing relatives or terminating a pregnancy, or how to weigh up competing rights to (not) know when they are in conflict (Clarke and Wallgren-Pettersson 2019). They will often be considered as issues of autonomy, beneficence or non-maleficence (Beauchamp and Childress 2019). Here, we wish to focus on a somewhat different set of concerns relating to the relatively neglected fourth principle, justice. While much scholarship is devoted to analysing what constitutes justice in healthcare, focussing on concepts such as the allocation of resources on the basis of need and how to respect equity or equality, much work is still needed to understand how these considerations may impact on practice.

To the extent that the promises of genomics work out, there are reasonable grounds for concern that the benefits will be available to some more than others, probably on the basis of wealth, social privilege and other advantages. Such inequity in access to the benefits of new technologies are regular features of other new technologies (Pappaioannou 2021) and indeed of healthcare in general (Tudor Hart 1971).

Given this background, how should professionals working in medical genetics and genomics respond to this potential for injustice? How can we work to ensure—or at least to make it more likely—that genomics is implemented in such a way that it does not exacerbate pre-exisiting inequities? Ideally, going further, we would also wish to consider how we might use this moment to reverse social injustices that already exist.

We raise these questions for consideration by grouping them in six domains. This approach aims to contribute to our understanding how the injustices operate in concrete settings, as a step towards mitigating these problems. We build on the distinction emphasised by Shklar between the nature of the concepts of justice and of injustice, with the concept of ‘justice’ being abstract while ‘an injustice’ refers to a more concrete and specific instance or experience of suffering from the absence of justice (Shklar 1990).

Some injustices are experienced at the personal, individual level and some at the level of the group. The impact of clinical genetics services has often been assessed at the level of the individual, using the tools of questionnaire design from psychology. While that can be helpful, we need in addition to hear people’s stories and to assess the collective impact of new developments on groups and the broader social consequences of new technologies and new policies. Contributions from the social sciences and humanities offer valuable insights in rendering such stories.

Furthermore, we must also hold in mind the past misuses of genetics. We cannot simply acknowledge the evil Race Hygiene and Race Superiority projects of Nazi Germany and move on without considering the complicity with eugenics policies and racism elsewhere and at other times, including in Western Europe, North America, sub-Saharan Africa, East Asia and other regions of the world. We need to consider what forms the abuse of genetics (and genomics) might take across the world today.

## Six aspects of genetics and genomics

We first consider medical genetics as one element within healthcare, constrained by the same factors leading to unjust outcomes as operate in medicine as a whole. Accessible and affordable care should be universally available through robust institutional arrangements, as is the case in most developed countries. Barriers to such high-quality care can operate at many levels, from the legal basis of health insurance to the very concrete setting of two individuals and their micro-interactions within the clinic, where the professional may fail to extend to the patient the full respect s/he is due. Many factors may feed into this, including ethnicity/race, caste, social class, or simply a personal antagonism. Other relevant factors may include over-stressed healthcare systems, where staff are exhausted and under-equipped, at least within the healthcare accessed by the poor and disadvantaged. These make it more difficult for staff to behave with the proper courtesy that we and they owe everyone, whether or not they are patients, but we owe this courtesy and respect especially to our patients.

The legal basis of health insurance operates in a country-specific fashion. One type of disadvantage, partially addressed in some countries, relates to the lifetime disadvantage experienced by those at increased risk of disease, or perhaps already affected by it, because of their genetic endowment. Institutional discrimination in healthcare and health insurance can then compound the difficulties resulting from natural processes.

We then turn to the area of data management and societal discrimination against groups on the basis of genetics. One area where injustice has already been well recognised in genomic healthcare arises from the over-representation of groups of European origin in population genetic studies and databanks. This makes the inference from such data for the interpretation of test results from other ethnic groups less reliable.

A third topic to be discussed is the way in which gene action and, consequently, the susceptibility to adult-onset diseases can be shaped by experiences in intrauterine life and early childhood. This area, now recognised as epigenetics, was opened up by David Barker (Barker et al. 1989) with his interpretation of lifetime patterns of disease as the result of ‘foetal programming’. Further work on these processes has shown how unjust social arrangements can perpetuate disadvantage in transgenerational cycles.

One area of specific importance in genomics is the mind. What will we learn about ourselves through neuropsychiatric genomics, and how may this shape psychiatry and impact on other aspects of society? How do we address the possibility of self-fulfilling prophecies in genetic testing within psychiatry? And are there topics we should not even explore?

While genomics within medicine has so far largely been applied as a technology to increase the efficiency of identifying single gene factors in disease, it has the ambition to address large-scale, genome-wide interactions and effects that may shape disease. The claims made by enthusiasts about this area are wide-ranging and often over-ambitious, potentially made with an eye to commercial success rather than clinical utility or even scientific validity. How should we as a community address these narratives when they are in competition? How can the public health best be protected and promoted, in a manner that respects efficiency and equity? How can we resist the over-eager application of genomic testing of individuals to problems that are more effectively and more cheaply addressed by the more traditional, collective responses of public health medicine cooperating with government and industry?

Finally, there are questions about the role of genetics and genomics within population screening programmes in relation to decisions about reproduction, such as prenatal screening in foetal life and the screening of healthy adults for genetic carrier status of recessive disorders. What is appropriate for society to promote? If harm may result from the promotion of screening, how may this be challenged or mitigated?

- (i) Social interaction and social structure

Access to health care has long been subject to systematic inequity, as famously explored by Julian Tudor Hart, a doctor in an area of South Wales then heavily industrialised (Tudor Hart 1971). While a state can provide universal health care, or ensure that all have access through social insurance schemes, it is more difficult to ensure that all can in practice access what has been made available. There remain many states that fail to ensure that all have even minimal access.

For disadvantaged individuals and groups in the population to have ready access to genomics services requires that they first be supported to seek medical attention and then to take up the offer of genomic investigation. When patients or whole families lack access to the internet or transport, are subject to poverty, disability and/or disease, or cannot attend a remote clinic without loss of a day’s pay, they are much less likely to access healthcare at all, let alone the new genomic technologies. Even if they do access genomics, they will be less likely to seek updates if their situation changes or a genetic variant is reinterpreted. We need to ensure that such people are effectively supported to access and maintain contact with health care systems. This requires public effort through the state as well as personal effort on the part of practitioners working to enable their patients—even their reluctant or impoverished patients—to engage with healthcare.

There will often be interactions and intersections between disadvantage and group membership, most often ethnicity. When markers of ethnicity coincide with cultural difference, as with customary consanguinity, there is a potential for disadvantage to be compounded by the experience of racism and social discrimination, even within healthcare settings. As practitioners, we have an obligation to be sensitive in how we behave towards members of disadvantaged groups. Furthermore, collective processes of disapproval also operate in response to cultural difference, as with antagonism to the practice of customary consanguinity. Attributions of guilt and blame can be heard not only at the individual level but and also, importantly, at the broader, societal and political level. This is a form of group discrimination acted out at the level of personal micro-interactions as well as in the public domain.

The same approach applies in relation to physical and intellectual disability as well. Judgemental attitudes held by practitioners towards those born with specific conditions can be very damaging. This also extends to judgemental attitudes held by practitioners about—i.e. against—those parents who choose either to terminate or to continue a pregnancy in which the foetus is likely to become a child affected by a disability (depending on the worldview, or even prejudice, of the healthcare professional).

There is also a question about investment in genomics and related technologies, including new, gene-based ‘rational’ therapies. How can the decisions about the allocation of resources to research and development, especially the step from the laboratory into translational medicine, be managed fairly between common disorders and rare diseases, and among the rare diseases? The decisions will have to take account of what is technically feasible but the interests and activities of particular lobbying groups will need to be understood from a broader perspective.

- (ii) Use and misuse of genomic data

Against the backdrop of personalised and precision medicine, which seeks to tailor prevention, diagnosis, and treatment more closely to the individual, genomic medicine becomes data medicine. Mining data from different sources, including genome sequence data, patient records, and lifestyles, and then linking it, opens up multiple new challenges, even within a jurisdiction such as the EU that has strong systems for control of personal data and protections against its misuse. What is now an adequate balance between data-driven patient monitoring on the one hand, and excessive health surveillance on the other? How should genomic and health data be collected and how should it be shared between clinical, academic, commercial and other settings? How can we minimise the risk of inappropriate discrimination against individuals or groups (Lipphardt et al. 2021), or other harms from misapplication of the data? When research is proposed that would examine the experiences of under-served groups, they will need to be engaged in ways that do not increase stigma, and patients should not feel pressured into contributing to research. Trustful relationships need to be established in which the research can be expected to benefit the community.

A sharp distinction has been drawn between health or genomic data and other types of personal information. Can this be justified, when so much information is available about individuals that may be relevant to their health? How can we enable data sharing, to promote the accurate interpretation of genomic investigations, while maintaining respect for genetic privacy and the control of personal data? These are important issues that need to be weighed with great care.

- (iii) Epigenetics, deprivation and the life course

Major differences have been observed between the prevalence of some common, degenerative disorders in different populations, such as the high incidence of Type 2 diabetes mellitus (T2D) in indigenous groups in North America, Australia and in some South Asian populations. One explanation proposed to account for such differences was James Neel’s idea of a ‘thrifty gene’, selected for by recurrent famines that predisposed to insulin resistance (Neel 1962). Another explanation invoked David Barker’s concept of ‘fetal programming’, in which a foetus sets its future metabolic pattern on the basis of its nutrient supply in utero, so that a deprived foetus would cope better with periods of starvation in later life but, in the presence of ample food supply, would be more likely to develop T2D or coronary artery disease (Barker et al. 1993). Since the early debates, it has become clear that molecular mechanisms exist in mammals that are able to account for such phenomena, although hard proof of their role in human disease is more difficult to obtain. The phenomena to be explained have also changed in important ways, so that transgenerational effects have been proposed, with grandpaternal starvation having delayed consequences for disease in their daughters’ children (Pembrey et al. 2006, 2014). As an important mechanism underlying disease and inequity, it has been relatively neglected (Räisänen et al. 2006).

These epigenetic processes would not be the cause of injustice but could, in the setting of poverty and the chronic distress resulting from colonialist oppression, lead to poor health outcomes and thereby reinforce the disadvantage experienced by these populations. We must not forget the brutality of the colonial oppression of indigenous groups and how it has continued at least until the recent past, especially in Australia (Allam and Evershed 2019). The choices made by public health authorities and politicians, as to what explanatory model of the biology to adopt to account for entrenched, cross-generational disadvantage in indigenous communities, has profound policy implications for health and social care (McDermott 1998): regarding it as genetic—caused by Neel's thrifty gene—could lead to a sense of inevitability and so inaction. In contrast, Barker's concept of foetal programming could generate the energy to transform the education, nutrition and healthcare for girls and young women in impoverished, especially indigenous, communities. Such research into the epigenetics of degenerative disease will also be relevant to migrant communities, as with those of South Asian ancestry in the UK, where there is an increased incidence of T2D but the complex interactions of genes, environment and life-course that underlie this are still not adequately understood.

Although political change is clearly necessary to make progress in these settings, biomedical researchers have the opportunity to contribute. There is the potential not only to explain biological processes that compound social disadvantage but also to develop our understanding of epigenetics with the aim of working to reverse these damaging consequences. However, while explanations for disease may emerge, we must recognise that prediction and intervention may remain elusive. The correlation between adverse risk factors for disease and social categories means that social categories are likely to continue to be used to make judgements about disease risk, with a dangerous potential for self-fulfilling predictions. Despite these difficulties, anything that might enable us to interpret and then interrupt these transgenerational cycles of social disadvantage, (nutritional) deprivation and disease would be enormously worthwhile (Meloni and Müller 2018; Dupras et al. 2019).

- (iv) Contentious applications of genetic testing in mental health and non-disease traits

The case for genetic research into mental ill health is strong. Indeed, it is already well established that susceptibility factors may be shared by different categories of psychiatric disease and it is hoped that research into this will generate important insights into disease mechanisms, which may assist the process of developing new and more effective treatments. It is also hoped that the use of genetic test results on patients will lead to the better selection of the most appropriate treatment for the individual, although these hopes remain as mere hopes, at least for now, and they are open to being ‘hyped’ and oversold.

As well as possible benefits, however, there is also a potential for the misapplication of genetic testing in the context of mental health, with harms caused to vulnerable individuals and groups. The use of ‘genomic prediction’—such as seeking to identify family members who share a patient’s susceptibility to mental illness—would inevitably induce anxiety, which in itself could lead to stress and contribute to the onset of disease. This possibility, that genetic prediction might lead to a self-fulfilling prophecy of mental illness, must be regarded as a serious contra-indication to such cascade testing in clinical practice. We need a broad discussion as to the settings, indications and supports required for the use of genomic information in mental health and psychiatric contexts. What would be appropriate if testing was based on well understood disease mechanisms, indicated predispositions of high penetrance, and opened the possibility of highly effective interventions for those found to be affected or at high risk? Given that most genetic markers for mental illness are only weakly associated with a phenotype—they are ‘polygenic’ markers of low penetrance—when, if at all, might they be helpful in clinical practice? What is the potential for social harms to result from genetic testing for susceptibility to mental illness, not only within the clinical setting but also in access to employment, education or immigration?

The potential problems of three different areas need to be considered, although they will overlap. One is the use of genetic testing to confirm a diagnosis. Given that a person’s genetic constitution is much the same throughout their life, and a psychiatric condition will often be episodic, this creates some difficulty in its utility as a tool for diagnosis (which is not the same as recognizing a susceptibility). Another potential application arises within a family context, as an offer of testing that follows the cascading of risk information through the kinship. This would amount almost to the pretence that psychiatric disease is determined by single genes of major effect, instead of its inheritance being highly complex. Finally, there is susceptibility testing as a population screening test (using polygenic risk scores), which will often be in the absence of any overt psychiatric symptoms. Given the difficulties with genotype-based prediction of phenotypes—so much more difficult than the search for the genetic basis of a clear clinical diagnosis—and especially the problem that some genetic variants are associated (weakly) with more than one distinct psychiatric phenotype, population screening must be regarded as even more problematic than attempts at diagnosis or family cascade testing.

Other aspects of the individual have also received much attention in psychology, especially intelligence. Indeed, intelligence is one of the most socially valued traits. However, research into intelligence has a long historical legacy of over-emphasis on hereditary factors leading to a deterministic approach that misrepresents what is known about intelligence (Gould 1981). This misrepresentation downplays the ability to modify traits through environmental interventions. The wilful misunderstanding of the concept of heritability—not recognising that it is not a fixed entity but varies with population subgroups and with circumstances—has been used politically to undermine environmental enrichment programmes for children in disadvantaged communities.

Intelligence has raised strong feelings and commitments among researchers. Experts, who are well aware that polygenic scores for IQ can account for only a small fraction (of the order of 10%) of the variance in IQ (Plomin and von Stumm 2018), nevertheless advocate the use of such testing. They recognise that such applications are fraught with societal hazards but this does not lead them to direct their research efforts to topics where they are less likely to cause harm (von Stumm and Plomin 2021). The application of genomic studies of IQ to the genetic testing of individuals for their ‘IQ potential’ is already available commercially, despite the lack of any clinical utility, and despite the likelihood of the deliberate misuse of such measures. Indeed, it is available in reproductive settings, as for preimplantation genetic testing (Palk et al. 2019). In other contexts, testing could readily be used to the disadvantage of those with ‘less good’ results, e.g. if such tests were used in the selection of applicants for education or employment, using genotype instead of phenotype.

Moving from individuals to social groups, as defined by ancestry or ethnicity, the comparison of their intelligence (as measured by IQ tests) or of their IQ-related genetic variants is likely to generate complex data that can easily be exploited in the cause of racism and with the aim of sowing discord. Indeed, this abuse has occurred repeatedly over many decades. Given this, it is paramount to involve the relevant communities in discussions on such research and assess whether it could lead to worthwhile outcomes if, for example, it led to a renewed focus on the environmental factors exacerbating community problems. There is no medical benefit to such research and the risks of pursuing it appear likely to outweigh the interest of the findings. Professionals in medical genetics have a particular responsibility to attend to public discussions on these topics and to correct erroneous arguments when they arise.

- (v) Genomics and the individualisation of responsibility for health as a distraction from basic health care

Disease prevention by health promotion can be approached collectively or individually. A key function of public health systems is to determine what aspects of health promotion are best tackled through collective action, and what by the decisions of individuals. While the benefits of collective public health measures will generally accrue to all members of society, those with advantages of wealth or status are more likely to purchase health screening technologies, healthy (but expensive) food, gymnastic club membership and other interventions that depend upon the actions of individuals. A focus on the individual can therefore undermine the moral and political case for commitment to collective action. The balance between individual and collective responsibility for disease prevention is of crucial importance in maintaining some equity in the provision of healthcare.

One way in which the individualisation of health care is promoted is through the sale to the general public, often DTC, of polygenic risk score testing (‘genome profiling’), for application in lifestyle advice. However, there is no evidence of clinical utility to support the clinical application of polygenic risk scores (PRSs), although this might change. Furthermore, the unwarranted use of PRSs is likely to trigger additional demands on medical services by those who have purchased the tests. The imposition of such opportunity costs on public health care systems would reduce the resources available for the appropriate care of all, especially the poor or otherwise disadvantaged, who have greater health care needs but are less likely to have sought PRS testing. Overviews of the application of PRS approaches to public health, even from enthusiasts, are cautious or critical (Polygenic Risk Score Task Force of the International Common Disease Alliance 2021).

One very real potential danger is that someone with a strong family history of a complex disorder, such as a familial cancer, may arrange testing through a DTC company for a range of disease susceptibilities. If they are given a low-ish or normal risk for the cancer, they may then fail to seek a full clinical genetics assessment. Their Mendelian risk may then not be identified until too late, as it is unlikely to be detected on PRS testing.

Genome-wide association studies (GWAS) have a valid role in research if their limitations are understood. These limitations include their use of common (polymorphic) variants, which are widespread and therefore necessarily’ancient’, so that they must not have been subject to strong, net negative or positive selection. They are blind to more (evolutionarily) recent, rare variants of greater effect, which are known to contribute to many disease-related complex traits (Young 2022), and our knowledge of how the common polymorphisms modify risk is often insufficient to be clear how independent they are of mediating physiological, environmental or life-course factors as well as gene–gene interactions. They are far from being robust enough to use in clinical practice (Wald and Old 2019; PHG Foundation 2021; Penders and Janssens 2021). Furthermore, the variants included in studies are predominantly those found in populations of, European ancestry (Martin et al. 2019). This means that polygenic risk profiles of individuals, derived from GWAS data, are of less applicability to individuals from non-European populations or where their ancestry shows admixture, perhaps being drawn from European and non-European populations.

Attempts to promote behaviour change in response to information about an individual’s genome-based ‘health risk profile’ have generally been ineffective (Hollands et al. 2016). Even if health promotion could be made effective, how could the combination of ‘genome profiling and behaviour change’ operate without exacerbating inequity? ‘Nudge’ has been proposed as a way of promoting appropriate behaviour change but would exacerbate the neglect of more effective and more equitable, collective approaches to public health. This focus on the individual’s behaviour would distract health services and politicians from the benefits of collective approaches to tackling poor public health, and thereby increase pre-existing inequities. We have no reason to trust that individualised approaches to health behaviours will succeed any better in the future than they have in the past, while these approaches threaten to undermine collective approaches to reducing everyone’s risk of disease. Professionals who understand both the genetic science and the limits of its applicability to healthcare need to speak out loudly against ill-informed and uncritical optimists and enthusiasts, who seek to profit from the misapplication of these methods in healthcare or even more broadly in the general population.

Enthusiasts have suggested some additional uses for PRS in tackling the common, complex diseases. Currently research is ongoing into whether PRS might be useful in a clinical setting e.g. as an additional tool to 'fine tune' monitoring or treatment decisions in families affected by monogenic subforms of common disorders, such as hereditary cancers. One suggested application is their use in population screening programmes; however, there is currently no evidence to support their use in such programmes. Another potential application is in comparing IVF embryos in a form of preimplantation genetic testing for disease susceptibility. Suggestions that this might be appropriate have been made on very weak grounds and have been firmly rejected as both impractical (because they are based on flawed science) and unethical (Turley et al. 2018; Forzano et al. 2021; Johnston and Matthews 2022; Nature 2022).

- (vi) Genomics, population genetic screening and reproduction

There are important social and ethical differences between societies in how they tackle the issues arising in population screening programmes relating primarily to reproduction: antenatal screening and carrier screening for recessive disease.

These carrier screening and antenatal (i.e. foetal) screening programmes are shaped by many factors, including the geographical distribution of genetic disease, the incidence of disease and the costs of treatment or prevention. Low-income countries may feel pressured to introduce and promote carrier screening programmes for specific conditions, in conjunction with prenatal diagnosis and the termination of affected pregnancies (e.g. for beta-thalassaemia) so as to contain the cost of health care. For such a country to afford treatment for those affected, the numbers born each year need to be low. This could be seen as imposing moral hazard on communities that are already disadvantaged. Something similar could arise in high income countries, once expensive, rational therapies have become more widely available for at least some rare disorders. Those at risk of having affected children may be pressured to accept prenatal diagnosis. After all, “Why should the taxpayer pay for the care of your (second) affected child, when you have declined to accept our offer of prenatal diagnosis?”.

The provision of genome-based tests in antenatal care introduces the potential for poor clinical practice if the offer of carrier screening or non-invasive prenatal genetic testing (NIPT) for chromosome anomalies is not of the highest quality. The knowledge base of midwifery and obstetric practitioners may be suboptimal, and the literature provided in support of these tests often glosses over important concepts, such as the test’s positive predictive value and the potential for misunderstanding of a ‘negative’ (reduced risk) result (Nuffield Council on Bioethics 2017). Choosing to decline testing, or being unable to afford testing if it is not included in social health care, could also result in a concentration of genetic disorders in more religious or less wealthy sectors of society. Parents may feel pressured into testing if they perceive a lack of social and medical support or experience an extreme burden when facilities are lacking for their affected child. This is another route through which social inequalities may be augmented in the context of genomics.

There is a plethora of economic and psychological factors that could motivate nation-states, corporations and individuals to consider setting up or participating in carrier screening programmes and prenatal genomic testing, often using NIPT. The desired outcomes of screening programmes, which must be recognised as setting their ethos and thereby shaping the experiences of the participants, will be evaluated differently by different parties. Public health agencies will assess a programme in a different way from a private company, as different costs are born by different agents and there are many indirect consequences of genetic conditions for society’s overall ‘balance sheet’. Individuals will consider many factors in weighing up their participation, with health promotion messages from the government or marketing from a private company both being relevant but set within a broader context of ‘life world’ factors. There are also the costs of new rational therapeutics developed for specific rare diseases to be considered alongside the other costs. Treatments may be so expensive that they may cost too much even for wealthy states to afford; this could motivate health services to ‘invest’ in systematic carrier screening and prenatal diagnosis, to limit the birth incidence of an ‘expensive’ disorder, as happened with carrier screening for thalassaemia in low-income countries.

What considerations should a practitioner bear in mind and act upon in the face of difficult clinical and family circumstances? It is a key principle of ethics in general, not only medical ethics or the ethics of clinical genetics services, that respect for the patient as a person, and for his or her autonomy, has to be paramount. While the practitioner may wish to promote a genetic screening programme, especially if the costs of treatment for a disorder can only be covered in that society if the birth incidence is low, these public health concerns must not overshadow and suppress the primary clinical obligation of caring for one’s patient. The individual practitioner must not subordinate their respect for the autonomy of the individual patient to their concern for the population. While that may be necessary in certain epidemics, this will certainly not apply in the context of genetic disorders.

## Conclusions

Those of us who are engaged in the application of genetics to healthcare have an obligation to be honest with ourselves, our patients and our communities. We can sing the praises of new genome-based technologies and we can hope that they will lead to more and better diagnostic or prognostic tools and therapeutic interventions. However, we must not exaggerate what can be achieved. We must not expect patients to participate even in observational research such as biobanks—and certainly not interventional research—for the benefit of humankind in the future, if it is to their detriment today. We must not put a glossy spin on the new test or the new treatment that is unjustified. We must not seek to apply new prenatal or predictive tests—to provide foreknowledge and to support reproductive decisions—if these are based on ‘optimistic conjecture’ about what the technology can deliver rather than firm knowledge. We must recognise that giving choices can be as burdensome as it is empowering, especially if the foreknowledge is questionable.

We have to achieve a balance between being hopeful and being realistic. With insufficient hope, one experiences a paralysis. But if false hopes are generated and then amplified, this leads to hopelessness, a sense of futility and resentment. A particular problem arises if the patient or family or support group commits to a goal and then fails to achieve it because the goal was never realistic. Being encouraged too far down the road to unreality by practitioners or researchers ‘hyping’ (i.e. exaggerating the benefits of) a possible treatment, is destructive; it stores up difficulties for the future. This will be especially so if the professionals have a conflict of interest. Perhaps for their career, their next publication, or their shares in a biotech company, they ‘need’ to recruit more research participants in a therapeutic trial and can do so by some ‘fine tuning’ of how they invite patients into the trial. How are such conflicts of interest discussed today in society—and in the ethics committee, and in the clinic? How should these conflicts of interest be addressed and managed? Not, we suggest, by ignoring the issue or pretending that transparency about a potential conflict of interest automatically neutralises it (as seems to happen in other areas of social and political life as well as healthcare).

If such an individual is a clinician, a biomedical scientist, a public servant (giving advice to government, perhaps) and/or an entrepreneur, the potential for judgements to be distorted by such conflicts of interest remains even when the potential conflict has been declared. There seems to be a naïve view that transparency dissolves such problems. Transparency can serve as a figleaf, a sop, to disarm critics while doing nothing to prevent inappropriate conduct. We in the world of medical genetics must prevent transparency being taken as a guarantor of trustworthiness. Addressing and engaging with such conflicts of interest may help reduce the potential for harm and injustices in research and care.

## Data Availability

This is a 'Perspective' article, not a research output, and no data were used.
